# An Investigation of Mortality Associated With Comorbid Pneumonia and Thrombocytopenia in a Rural Southwest Missouri Hospital System

**DOI:** 10.7759/cureus.67330

**Published:** 2024-08-20

**Authors:** Tabitha Ranson, Hannah Rourick, Rajbir Sooch, Nicole Ford, Nova Beyersdorfer, Kerry Johnson, John Paulson

**Affiliations:** 1 College of Medicine, Kansas City University, Joplin, USA; 2 Primary Care, College of Medicine, Kansas City University, Joplin, USA; 3 Mathematics, Missouri Southern State University, Joplin, USA

**Keywords:** inpatient mortality, hospitalization, rural, mortality, pneumonia and thrombocytopenia, thrombocytopenia, pneumonia

## Abstract

Background: Pneumonia places a significant burden on individuals and society, contributing to a substantial number of hospital admissions, emergency department visits, deaths, and healthcare costs each year. Comorbidities can greatly increase the risk of poor outcomes when associated with pneumonia. One comorbidity that has yet to be thoroughly researched is thrombocytopenia, which is known to play an important role in activating the immune response to infections. A decrease in platelet count may limit the immune response and consequently increase mortality in patients with pneumonia. The purpose of this study was to investigate whether comorbid thrombocytopenia and pneumonia are associated with poor outcomes.

Methods: This study was a retrospective cohort analysis comparing mortality rates among patients with comorbid thrombocytopenia and pneumonia, pneumonia without thrombocytopenia, and thrombocytopenia without pneumonia. Data were collected from Freeman Health System using International Classification of Diseases, Tenth Revision (ICD-10) codes from January 1, 2019, to December 31, 2021. ICD-10 codes for pneumonia and thrombocytopenia were extracted and stratified into three groups: those with both pneumonia and thrombocytopenia, those with pneumonia without thrombocytopenia, and those with thrombocytopenia without pneumonia. Mortality rates were then compared across the three groups.

Results: There were 4,414 patients admitted with pneumonia and 1,157 admissions for thrombocytopenia without pneumonia. Among the 4,414 patients admitted with pneumonia, 3,902 did not have thrombocytopenia, while 512 had thrombocytopenia. Of the patients without thrombocytopenia, 14% (3,902) expired. Among the 512 patients with thrombocytopenia, 43% expired. In the thrombocytopenia without pneumonia group, 11% (1,157) expired.

Conclusion: These results indicate a significant increase in mortality in patients with both pneumonia and thrombocytopenia compared to those with pneumonia without thrombocytopenia (an increase in mortality of 28.93% with a 95% CI: 24.50-33.36%, P < 0.0001). While pneumonia itself increases mortality compared to the general population, patients with both pneumonia and thrombocytopenia exhibit even higher mortality rates.

## Introduction

The nationwide yearly incidence of admissions for pneumonia in the United States is estimated to be 649 hospitalizations per 100,000 adults, making it a significant healthcare and economic concern [1]. In 2022, there were 1,542,000 emergency department visits and 41,309 deaths due to pneumonia in the United States [2]. The incidence and severity of pneumonia have led to increased costs for both individual patients and hospital systems. In the United States, where pneumonia is the eighth leading cause of death, the total cost of treatment has been estimated at seventeen billion dollars per year, with the average cost of hospitalization per patient due to pneumonia being $61,928 [3, 4]. These numbers demonstrate the significant burden pneumonia places on both society and individuals. Patients with pneumonia are exceedingly prone to hospital readmission, with rates up to 20% in elderly populations [5]. As with many other diseases, when combined with comorbidities, the body’s ability to recover from pneumonia can be significantly impacted. Short- and long-term effects of pneumonia include readmission, cardiac events, cognitive impairment, and death [6]. Understanding factors that contribute to poor outcomes can inform treatment strategies to decrease secondary effects and mortality.

One possible factor contributing to poor outcomes in pneumonia is thrombocytopenia, defined as a platelet count of less than 150,000 per microliter of blood [7]. While platelets are primarily involved in hemostasis, they also have key interactions with both the innate and adaptive immune systems. Platelets utilize both direct and indirect pathways to clear bacterial pathogens from the body. Directly, platelets bind bacterial pathogens through GP1ba, GPIIb/IIIa, integrin surface receptors, and toll-like receptors, which ultimately leads to phagocytosis, intracellular killing, mechanical removal of bacteria, neutrophil extracellular trapping, and complement-induced killing [8]. When activated, platelets act indirectly by releasing granules containing chemokines that increase vascular permeability and leukocyte extravasation [8].

In addition to their role in bacterial infections, platelets have also been shown to impact the response to viral infections. A decreased platelet count is correlated with disease severity in viral infections [9]. Platelets appear to affect RNA translation, directly impacting viral replication [10]. Given the important role platelets play in both bacterial and viral infections, understanding the relationship between platelets and the immune response, and their correlation with patient outcomes, is valuable for patient care.

Given the crucial role of platelets in clearing bacterial and viral pathogens, patients with low platelet counts may have a reduced ability to fight these infections. Patients with both pneumonia and thrombocytopenia admitted to the ICU have been found to have a higher need for invasive mechanical ventilation and increased rates of sepsis [11]. Efficient recognition of this comorbidity is useful in preparing for possible complicated clinical courses and aggressive treatment plans. Therefore, patients with conditions leading to low platelets or those taking medications that decrease platelet function may be at increased risk for poor outcomes with pneumonia.

Previous studies have been limited by only examining the association between thrombocytopenia and pneumonia through the lens of specific pathogens, a singular pneumonia type, or patients within distinct units of the hospital and were often limited by small population sizes [12, 13]. This study sought to analyze a larger, more generalized population of patients within an entire hospital setting. It aims to fill the gap in current research by determining whether those with a combination of thrombocytopenia and pneumonia are at an increased risk for poor outcomes. This information is relevant to providers as it can inform treatment strategies to mitigate the risk of poor outcomes.

The purpose of this study was to investigate thrombocytopenia as a risk factor for poor outcomes in hospitalized patients with pneumonia. Given the various roles of platelets in activating the immune response to both bacterial and viral pathogens, we hypothesized that thrombocytopenia would be associated with increased mortality in patients with pneumonia compared to those without thrombocytopenia. To evaluate this hypothesis, a retrospective observational study was conducted by extracting the International Classification of Disease, Tenth Revision (ICD-10) codes for the diagnoses of pneumonia and thrombocytopenia in hospitalized patients. We then evaluated the differences in mortality rates among these populations and their comorbid states.

## Materials and methods

Data collection

This retrospective observational cohort study used patient data collected from electronic medical records at Freeman Health System (FHS), a not-for-profit hospital located in Joplin and Neosho, Missouri, with 410 and 25 licensed beds, respectively. Data were extracted from discharges between January 1, 2019, and December 31, 2021. All participants were over 18 years old, with no other restrictions regarding demographics. Due to the retrospective nature of the study, informed consent was not required.

Statistical analysis

**Figure 1:**
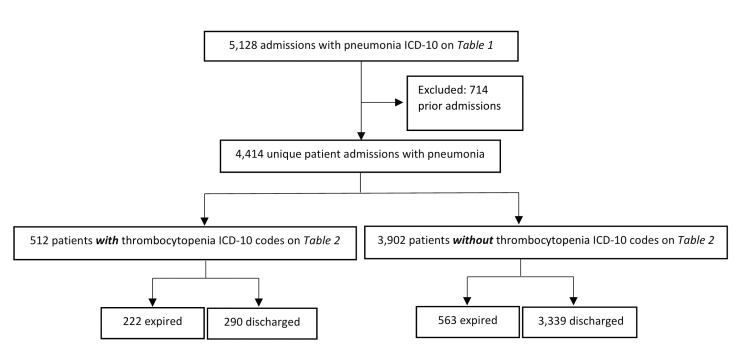
Pneumonia population group numbers separated into categories with and without thrombocytopenia diagnoses. ICD: International Classification of Diseases.

**Figure 2:**
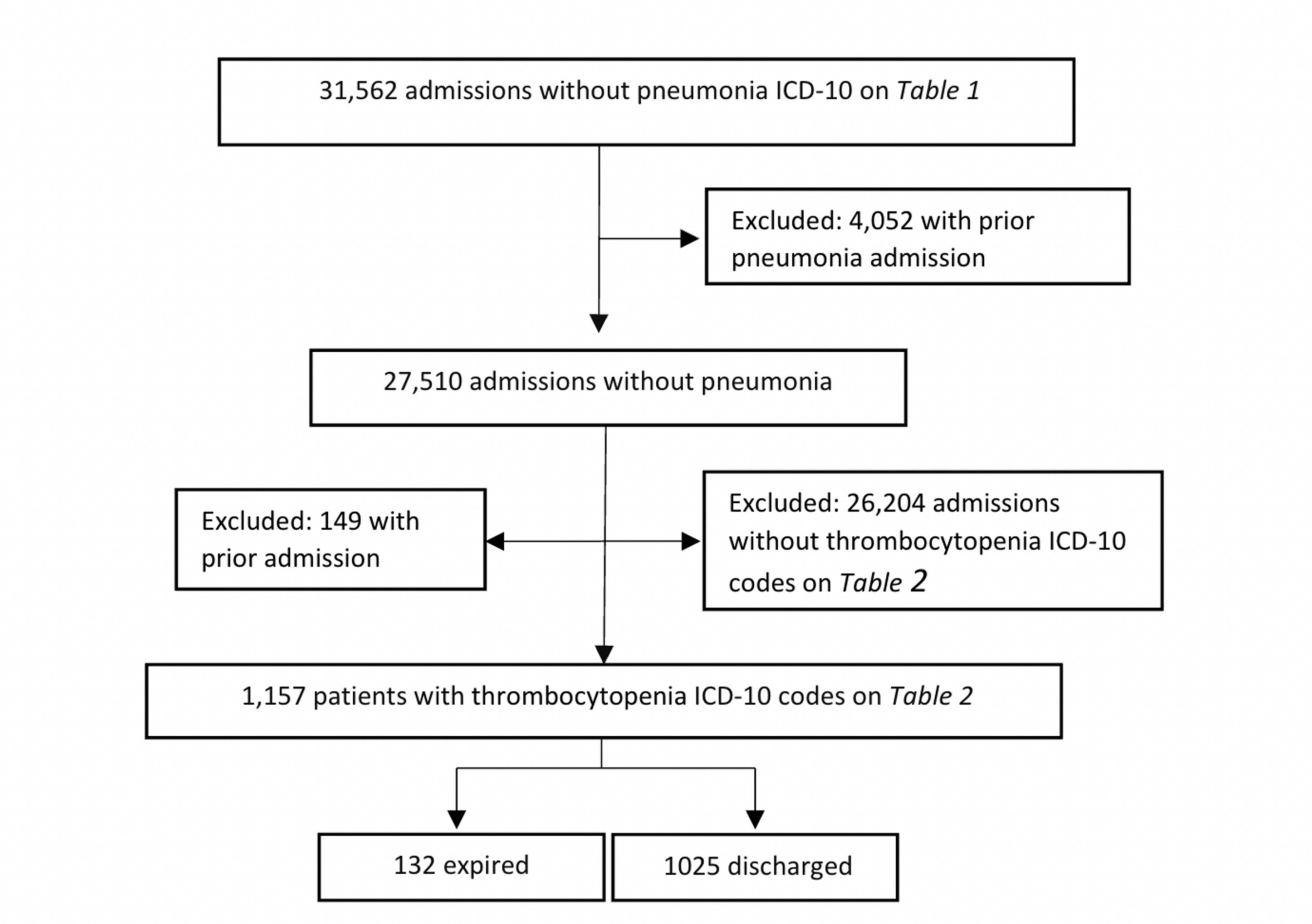
Population group numbers of those without pneumonia separated into categories with and without thrombocytopenia diagnoses. ICD: International Classification of Diseases.

Data for our chosen patient populations were extracted using ICD-10 codes to identify patients with or without diagnoses of pneumonia and thrombocytopenia (Tables 1, 2). The initial sample of patients with a pneumonia diagnosis consisted of 5,128 patients. Exclusions were made in all groups if patients had prior admissions, leaving an initial sample size of 4,414 unique patients with a pneumonia diagnosis. Within that sample, 512 patients had both pneumonia and thrombocytopenia (P1). Additional stratification identified 3,902 patients with pneumonia but without thrombocytopenia (P2) (Figure 1). The initial sample of patients without pneumonia was 31,562, of which 4,052 were excluded due to prior admission with pneumonia. Of the remaining 27,510 patients in that sample, 1,157 were identified as having thrombocytopenia without pneumonia (P3) (Figure 2). Statistical analysis determined proportion differences between populations using two-sample proportion summary hypothesis tests. Individual sample proportions were analyzed using Wald’s method. Data were considered significant with a P-value <0.05, and a 95% confidence interval (CI) was used in the analysis.

**Table 1:** ICD-10 codes used to identify the initial pneumonia sample ICD: International Classification of Diseases.

ICD-10 Code	Diagnosis
J1000	Influenza due to other identified influenza viruses with unspecified types of pneumonia
J1001	Influenza due to other identified influenza viruses with the same identified influenza virus pneumonia
J1008	Influenza due to other identified influenza viruses with other specified pneumonia
J1100	Influenza due to an unidentified influenza virus with an unspecified type of pneumonia
J1108	Influenza due to an unidentified influenza virus with specified pneumonia
J120	Adenoviral pneumonia
J121	Respiratory syncytial virus pneumonia
J122	Parainfluenza virus pneumonia
J123	Human metapneumovirus pneumonia
J1281	Pneumonia due to SARS-associated coronavirus
J1282	Pneumonia due to coronavirus disease 2019
J1289	Other viral pneumonia
J129	Viral pneumonia, unspecified
J13	Pneumonia due to *Streptococcus pneumoniae*
J14	Pneumonia due to *Hemophilus influenzae*
J150	Pneumonia due to *Klebsiella pneumoniae*
J151	Pneumonia due to *Pseudomonas*
J1520	Pneumonia due to *Staphylococcus*, unspecified
J15211	Pneumonia due to methicillin-susceptible *Staphylococcus aureus*
J15212	Pneumonia due to methicillin-resistant *Staphylococcus aureus*
J1529	Pneumonia due to other *Staphylococcus *species
J153	Pneumonia due to *Streptococcus*, group B
J154	Pneumonia due to other streptococci
J155	Pneumonia due to *Escherichia coli*
J156	Pneumonia due to other gram-negative bacteria
J157	Pneumonia due to *Mycoplasma pneumoniae*
J158	Pneumonia due to other specified bacteria
J159	Unspecified bacterial pneumonia
J168	Pneumonia due to other specified infectious organisms
J17	Pneumonia in diseases classified elsewhere
J180	Bronchopneumonia, unspecified organism
J181	Lobar pneumonia, unspecified organism
J188	Other pneumonia, unspecified organism
J189	Pneumonia, unspecified organism
J84116	Cryptogenic organizing pneumonia
J851	Abscess of the lung with pneumonia
J95851	Ventilator-associated pneumonia

**Table 2:** ICD-10 codes used to identify thrombocytopenia populations ICD: International Classification of Diseases.

ICD-10 Code	Diagnosis
D6942	Congenital and hereditary thrombocytopenia purpura
D6949	Other primary thrombocytopenia
D6959	Other secondary thrombocytopenia
D696	Thrombocytopenia, unspecified
D7582	Heparin-induced thrombocytopenia (HIT)

## Results

The sample with pneumonia and thrombocytopenia had a mortality rate of 43.36% (95% CI: 39.07-47.65%), significantly higher than the population with pneumonia but without thrombocytopenia (Table 3). Both populations with a pneumonia diagnosis had a significantly higher mortality rate than the population without a pneumonia diagnosis. The sample with both pneumonia and thrombocytopenia had a 31.95% (P < 0.0001; 95% CI: 27.28-36.62%) higher mortality rate than the population without pneumonia, while the group with pneumonia but without thrombocytopenia had a 3.02% (P < 0.0088; 95% CI: 0.88-5.16%) higher mortality rate than the group without pneumonia but with thrombocytopenia (Table 4).

**Table 3:** Mortality comparisons of all sampled populations P1: Population with pneumonia and with thrombocytopenia; P2: Population with pneumonia and without thrombocytopenia; P3: Population with thrombocytopenia and without pneumonia; CI: Confidence Interval.

Sample Populations	Mortality	Sample Proportion	Lower 95% CI	Upper 95% CI
P1	222 of 512	43.36%	39.07%	47.65%
P2	563 of 3902	14.43%	13.33%	15.53%
P3	132 of 1157	11.41%	9.58%	13.24%

**Table 4:** Two-sample proportion comparisons of mortality rates P1: Population with pneumonia and with thrombocytopenia; P2: Population with pneumonia and without thrombocytopenia; P3: Population with thrombocytopenia and without pneumonia; CI: Confidence Interval.

Comparison	Mortality Sample 1	Mortality Sample 2	Sample 1 vs Sample 2	Lower 95% CI	Upper 95% CI	P-value
P1 vs P2	222 of 512	563 of 3902	28.93%	24.50%	33.36%	<0.0001
	43.36%	14.43%				
P1 vs P3	222 of 512	132 of 1157	31.95%	27.28%	36.62%	<0.0001
	43.36%	11.41%				
P2 vs P3	563 of 3902	132 of 1157	3.02%	0.88%	5.16%	0.0088
	14.43%	11.41%				

## Discussion

The purpose of this study was to determine if comorbid pneumonia and thrombocytopenia are associated with poor outcomes in hospitalized patients. Previous studies have demonstrated that hospital-acquired and ventilator-associated pneumonia are major risk factors for mortality in settings such as the ICU and found that approximately one out of three patients hospitalized with community-acquired pneumonia died within one year [1, 14]. Other studies have indicated that thrombocytopenia is linked to adverse outcomes in patients with community-acquired pneumonia and that rising platelet counts are indicators of increased survival while falling platelet counts are prognostic of poor outcomes [11, 15]. Our results indicate that patients with concurrent pneumonia and thrombocytopenia have a higher mortality rate than those diagnosed with pneumonia without thrombocytopenia and those with thrombocytopenia without pneumonia. This association may be related to the role of platelets in immunity. While platelets are well-known for their role in coagulation, they also play an important role in aiding immunity during infection. During an infection, inflammatory cytokines and chemokines induce platelet activation. Platelets then release granules containing immunoregulatory proteins that can halt pathogen proliferation and even destroy some pathogens [8, 16-18]. Given the role of platelets in immunity, worse outcomes could be expected in patients diagnosed with comorbid pneumonia and thrombocytopenia.

Our results also indicated that patients with pneumonia but without thrombocytopenia had a significantly higher mortality rate compared to patients with thrombocytopenia but without pneumonia; however, the difference was found to be as low as 0.88%, as indicated by the confidence interval. Compared to the much larger significant differences found between the other comparison groups, this small difference may indicate that thrombocytopenia on its own may also be associated with inpatient mortality.

Our results suggest that thrombocytopenia has the potential to be an indicator of mortality risk in patients admitted with pneumonia in rural populations in Southwest Missouri. Identifying risk factors for poor outcomes in patients with pneumonia can inform providers of the level of intervention needed for these patients. This information can also assist both patients and their families in making decisions about life-saving versus supportive care in cases of severe pneumonia. The association found between thrombocytopenia, pneumonia, and poor outcomes may also indicate that those with thrombocytopenia have an increased risk of mortality when contracting pneumonia compared to the general population, suggesting a need to increase pneumonia prevention efforts in certain high-risk populations. Prevention efforts could emphasize pneumococcal vaccination or increased avoidance of exposure in immunosuppressed populations.

In addition to thrombocytopenia being a potential indicator of mortality risk in patients with pneumonia, thrombocytopenia on its own may be an ominous finding, as the mortality rate of thrombocytopenia without pneumonia was only slightly higher than that of pneumonia without thrombocytopenia. These findings are interesting, as pneumonia is historically considered a more severe disease. However, in ICU patients, thrombocytopenia has been found to be an independent variable for risk stratification, with platelet counts lower than 150,000 indicating increased mortality [19]. Another study analyzed the association between platelet counts and mortality in critically ill patients with tumors and found that both thrombocytopenia and thrombocytosis were associated with higher mortality rates [20]. These studies suggest that platelet levels may be associated with a worse prognosis in critically ill patients.

Limitations

The generalizability of our study is limited to people in rural communities in Southwest Missouri with pneumonia severe enough to require hospital admission. Currently, it is unclear whether the data linking thrombocytopenia to poor outcomes in patients with pneumonia can be applied to other geographic locations or to patients with pneumonia who are not admitted to the hospital.

Other important limitations of this study include potential bias and errors of imprecision. As this is a retrospective study, the population was not randomized, which may skew the magnitude of the results in a positive direction. Without the ability to randomize subjects, this data may not be representative of the population as a whole. Given the retrospective design, all variables were determined prior to initiating the study. Since the data were already reported, a lack of standardization could be a concern. The lack of standardized reporting makes the data vulnerable to errors, including underdiagnosed pneumonia, misdiagnosis of pneumonia, and possible lab errors indicating thrombocytopenia. Underdiagnosing pneumonia may negatively skew results, while misdiagnosis of pneumonia or lab error indicating thrombocytopenia could positively skew our results. A generalized lack of standardization for cutoff values of thrombocytopenia may also impact our results. Confounding factors, including age, gender, comorbid conditions, and disease severity, may also contribute to bias in this study, affecting the determination of pneumonia and thrombocytopenia as independent risk factors for mortality.

Given the retrospective study design, we cannot determine whether thrombocytopenia occurred before or after pneumonia. Information regarding temporality would more accurately inform the care of those with preexisting thrombocytopenia. The current data limits our ability to determine whether thrombocytopenia played a role in decreased immunity, possibly leading to pneumonia, or if thrombocytopenia occurred as a result of pneumonia. Understanding this cause-and-effect relationship could allow for more accurate guidance of treatments. If thrombocytopenia increases the risk of poor outcomes in pneumonia, then medical intervention to increase platelet counts could improve the disease course. However, if thrombocytopenia occurs as a result of the severity of pneumonia, it may indicate an ominous prediction regarding immune system function.

Another consideration is the type of pneumonia and the relative risk associated with thrombocytopenia. The methodology for this study used ICD-10 codes for several different types of pneumonia, including both bacterial and viral. Some evidence indicates a stronger role for platelets in the antibacterial immune response compared to the antiviral immune response [8]. Therefore, thrombocytopenia may increase the risk of mortality associated with bacterial infections compared to viral infections. The results of this study could be positively skewed based on the prevalence of bacterial versus viral pneumonia.

Recommendations

While our research indicates a link between thrombocytopenia and pneumonia, more research is needed to determine the specific role of decreased platelets in increased mortality. Future research may include studies examining the potential cause-and-effect relationship between the two diagnoses and how this relationship may pertain to those with preexisting thrombocytopenia. Confounding factors such as age, gender, comorbid disease, and severity of the conditions should also be explored as variables. Future evidence regarding the role of platelets in pneumonia-associated mortality can help inform healthcare providers as they determine the clinical course and medical interventions for their patients.

## Conclusions

With pneumonia being one of the most common causes of hospital admission and a significant risk to many populations, studying its outcomes and comorbidities is critical for treatment planning and mitigation. Our study indicates that the risk of poor outcomes is significantly higher in patients with pneumonia and comorbid thrombocytopenia compared to those diagnosed with pneumonia or thrombocytopenia alone. Interestingly, pneumonia without thrombocytopenia shows only a minimal increase in mortality compared to thrombocytopenia without pneumonia, demonstrating a potential link between platelet counts and patient mortality. Further studies are needed to investigate the cause-and-effect relationship between pneumonia and thrombocytopenia, which could further assist in treatment management. Additionally, larger and more varied populations should be studied to increase generalizability.
